# VMP1, a novel prognostic biomarker, contributes to glioma development by regulating autophagy

**DOI:** 10.1186/s12974-021-02213-z

**Published:** 2021-07-26

**Authors:** Wanzun Lin, Yun Sun, Xianxin Qiu, Qingting Huang, Lin Kong, Jiade J. Lu

**Affiliations:** 1 Department of Radiation Oncology, Shanghai Proton and Heavy Ion Center, Fudan University Cancer Hospital, 4365 Kangxin Rd, Pudong, Shanghai, 201321 China; 2 Shanghai Key Laboratory of Radiation Oncology (20dz2261000), Shanghai, 201321 China; 3 Shanghai Engineering Research Center of Proton and Heavy Ion Radiation Therapy, Shanghai, 201321 China; 4 Department of Radiation Oncology, Shanghai Proton and Heavy Ion Center, 4365 Kangxin Rd, Pudong, Shanghai, 201321 China

**Keywords:** Glioma, Prognosis, VMP1, Autophagy, Biomarker

## Abstract

**Background:**

Malignant glioma, especially glioblastoma, is a highly aggressive disease with a dismal prognosis. Vacuole membrane protein 1 (VMP1) is a critical autophagy-associated protein with roles in oncogenesis and tumor progression. However, the contribution of VMP1 to glioma development as well as its prognostic value has not been established.

**Methods:**

The expression of VMP1 and clinicopathologic data for 1996 glioma samples were collected from authoritative public databases to explore its prognostic value. Lentiviral CRISPR-Cas9 gene editing system was performed to deplete VMP1 expression. Apoptosis assays, cell cycle assays, colony formation assays, and EdU incorporation analysis were conducted to validate the biological function of VMP1. Transmission electron microscopy was used to determine the role of VMP1 in regulating autophagy.

**Results:**

VMP1 overexpression was associated with advanced disease and had a poor prognosis in patients with glioma. The depletion of VMP1 by CRISPR-Cas9 gene editing significantly inhibited cell proliferation, increased cell death, and induced cell cycle arrest. Mechanistically, VMP1 knockout blocked autophagic flux and thus sensitized glioma cells to radiotherapy and chemotherapy. Moreover, a nomogram model showed that VMP1 expression has high prognostic value for determining survival in glioma.

**Conclusions:**

Our results provide insights into the pathological and biological functions of VMP1, including its roles in promoting tumor growth and progression, and support its value as a new diagnostic and prognostic biomarker for glioma.

## Introduction

Malignant glioma, especially glioblastoma (GBM), has a dismal prognosis [1]. Despite extensive in vitro and in vivo studies, the complex heterogeneity of glioma hinders the development of novel treatment modalities [2]. GBM is the most aggressive type of malignant tumor, which is characterized by high mitotic activity, hypoxia, necrosis, inflammation, rapid cell proliferation, and high autophagic activity [3, 4]. Autophagy is a critical self-renewal mechanism conferring a malignant phenotype in glioma, leading to resistance to surgery, radiotherapy, chemotherapy, or immunotherapy [5–8]. Further studies on the pathogenesis of glioma are needed to develop advanced treatments and identify effective diagnostic and prognostic biomarkers.

Vacuole membrane protein 1 (VMP1) is an endoplasmic reticulum (ER)-resident and multi-spanning membrane protein. It was recently identified as an essential autophagy-related protein [9–11]. VMP1 expression can be induced by starvation or rapamycin, triggering the transformation of microtubule-associated protein 1 light chain 3 (LC3)-I to LC3-II and autophagosome formation in mammalian cells [12]. In contrast, the depletion of VMP1 substantially disrupts autophagosome formation by inhibiting the separation of autophagosome precursors from the ER [11]. VMP1 also plays important roles in interaction between the ER and other cellular components, including lipid droplets, Golgi, and endosomes [12, 13]. The mechanisms by which VMP1 modulates autophagy in mammalian cells have gradually been revealed; however, the functions of VMP1 in tumorigenesis and tumor development as well as its prognostic value are not clearly established.

In this study, we aimed to (i) explore the associations of VMP1 expression with clinicopathologic features in glioma, (ii) evaluate its prognostic value, (iii) elucidate the biological functions and underlying signaling pathways of VMP1 in the development and progression of glioma, and (iv) determine the role of VMP1 in modulating glioma autophagy.

## Materials and methods

### Data acquisition and processing

A total of 1895 samples with RNA-seq profiles and corresponding clinicopathological data were collected from the Chinese Glioma Genome Atlas (CGGA, www.cgga.org.cn; *n* = 929), The Cancer Genome Atlas (TCGA, http://cancergenome.nih.gov; *n* = 690), and Gene Expression Omnibus (GEO, accession number: GSE16011; *n* = 276) [14–16].

Additionally, 101 glioma specimens embedded in tissue microarray blocks (G6042) were obtained from Servicebio (Wuhan, China) and used to further evaluate VMP1 protein expression and prognostic value in glioma.

### Functional enrichment analysis

A gene set enrichment analysis (GSEA) was applied to compare gene set enrichment in the high and low-risk groups using the MSigDB Collection (c2.cp.kegg.v7.4.symbols.gmt). For each analysis, 1000 gene set permutations were used. The VMP1 expression level was used as a phenotype label. The standard for high and low VMP1 expression was determined based on the median value of VMP1 expression. The pathway enrichment of differentially expressed genes (DEGs) between VMP1 high- and low-risk groups was performed using the ‘ClusterProfiler’ package in R [17].

### Lentiviral CRISPR-Cas9 gene editing system

A CRISPR-Cas9 small guide RNA (sgRNA) lentiviral vector targeting human VMP1 was obtained from Genechem Co., Ltd. (Shanghai, China). Oligos corresponding to the sgRNAs were synthesized and cloned into the lentiviral CRISPR plasmid U6-sgRNA-EF1a-Cas9-FLAG-P2A-puro. The sgRNA sequences were as follows: control, CGCTTCCGCGGCCCGTTCAA; sgRNA1, GAGACGTGTAGCAATGAACA; sgRNA2, ATCTATGAAATGGCAGAGAA; and sgRNA3, ATGAACAAGGAACATCATAA. Lentiviral particles were produced in HEK293 cells and were collected via ultracentrifugation at 19,400×*g* at 4 °C for 2.5 h.

For the transfection experiment, 1 × 10^5^ LN299 cells were previously seeded into a 6-well plate and cultured overnight. The cells were transfected with 10 μL of lentiviral vector (5 × 10^8^ TU/mL; multiplicity of infection = 10) in 1 mL of media with 5 μg/mL polybrene. After 16 h, the supernatant was replaced with conventional culture medium and samples were continuously cultured for another 48 h. Thereafter, 3 μg/mL puromycin was used to screen the stable transfectants.

### Apoptosis assays

Apoptotic cells were determined using FITC Annexin V Apoptosis Detection Kit I (cat #556547; BD Pharmingen, BD Biosciences, Franklin Lakes, NJ, USA). In brief, LN299 were trypsinized, washed twice with the phosphate-buffered saline (PBS), and resuspended in 1× binding buffer. Thereafter, 100 μL of the solution was supplemented with 5 μL of FITC-annexin V and propidium iodide (PI) and incubated for 15 min at room temperature in the dark. The percentage of apoptotic cells was determined using a flow cytometer (CytoFLEX S; Beckman Coulter, Brea, CA, USA).

### Cell cycle assays

Cell cycle assays were performed according to instruction for flow cytometry using the PI/RNase Staining Buffer Kit (cat #550825; BD Pharmingen). Briefly, 1 × 10^6^ cells were collected and fixed with cold 70% ethanol at − 20 °C for 24 h. The fixed cells were washed twice and stained with PI/RNase Staining Buffer for 15 min at room temperature. Next, the stained cells were analyzed using a flow cytometer (CytoFLEX S; Beckman Coulter).

### Colony formation assays

For colony formation assays, 1000 cells were seeded in 6-well plates. After 14 days, colonies were fixed with 4% paraformaldehyde and stained with crystal violet staining solution (cat #C0121; Beyotime Biotechnology, Shanghai, China). Images and colony counts were obtained using a colony counting machine (GelCount; Oxford Optronix Ltd., Milton, UK).

### Cell immunofluorescence

Cells were previously cultured on confocal dishes (cat #FCFC016; Beyotime Biotechnology). On the following day, cells were fixed with 4% paraformaldehyde, washed with PBS three times, permeabilized with 0.1% Triton X-100, and blocked with 5% bovine serum albumin in PBS for 1 h. Then, cells were incubated with a phospho-S139 r-H2AX antibody (1:150) overnight at room temperature, with Alexa 488 goat anti-rabbit antibody (1:100) for 2 h at room temperature, and finally with DAPI staining solution (cat #C1005; Beyotime Biotechnology) for 5 min. Images were obtained using a confocal laser scanning microscope.

### Immunohistochemical (IHC) analysis

The experimental procedure for IHC has been described previously [18]. Briefly, the tissue microarray slides were analyzed via immunohistochemistry with anti-human VMP1 (1:200; cat #12978S, Cell Signaling Technology, Danvers, MA, USA), followed by a horseradish peroxidase (HRP) secondary antibody (cat #ab205718; Abcam, Cambridge, UK) and DAB. Images were obtained using a microscope (BX43; Olympus, Tokyo, Japan) at 200× magnification. The histochemistry score was applied to assess VMP1 expression.

### Transmission electron microscopy (TEM)

LN299 cells were previously fixed with 0.1 M cacodylate buffer and 2.5% glutaraldehyde for 2 h, trypsinized, and washed with cold PBS. Next, cells were treated with 1% osmium tetroxide and 0.1 M cacodylate. The fixed cells were dehydrated follow various concentrations of ethanol and then embedded in epoxy resin. Cell ultrastructures were observed, and images were obtained under the Hitachi TEM system at 80 kV.

### Western blot analysis

Briefly, LN299 cells were treated with RIPA buffer (cat #P0013B; Beyotime Biotechnology) containing protease inhibitors (cat #78425; Thermo Fisher, Waltham, MA, USA). After 10 min of incubation on ice, lysates were centrifuged at 15,000 rpm for 10 min at 4 °C to obtain the supernatant. The concentration of protein was determined using the BCA Assay Kit (cat #P0010S, Beyotime Biotechnology), and 20 μg of protein sample was separated via SDS-PAGE and blotted onto a polyvinylidene fluoride (PDVF, cat #FFP33, Beyotime Biotechnology) membrane. The PVDF membrane was then blocked with 5% bovine serum albumin (cat #ST023, Beyotime Biotechnology) in Tris-buffered saline with Tween-20 (TBST) for 1 h at room temperature and incubated with a VMP1 primary antibody (1:2000; cat #12978S, Cell Signaling Technology) at 4 °C overnight. On the next day, the PVDF membrane was incubated with the HRP-conjugated antibody (1:3000; cat #7074S, Cell Signaling Technology) for 2 h and washed three times with TBST. The bands were then visualized using a Bio-Rad system (Hercules, CA, USA) following the instructions.

### EdU incorporation analysis

An EdU incorporation assay was conducted using the BeyoClick EdU Cell Proliferation Kit (cat #C0078S; Beyotime Biotechnology) following the instructions. Briefly, cells were cultured with 10 μM EdU for 4 h at 37 °C/5% CO_2_. The cells were then fixed and permeabilized. After they were washed with PBS three times, cells were incubated with Click Additive Solution for 30 min at room temperature and fluorescent images were obtained using a confocal laser scanning microscope.

### Statistical analysis

Relationships between VMP1 expression and clinicopathological features were tested using the Wilcoxon test and visualized using R (version 3.6.0). Overall survival (OS) was compared between groups with high and low VMP1 expression by a Kaplan–Meier analysis. The median value of VMP1 expression was used as grouping criteria of patients. Univariate Cox analyses were performed to identify prognostic factors, and a multivariate Cox analysis was used to determine VMP1 expression as an independent risk factor for OS in glioma. Student’s *t*-tests were performed using GraphPad 7.0. *P* < 0.05 was considered statistically significant.

## Results

### VMP1 is overexpressed in glioma and is associated with an advanced stage

**Fig. 1:**
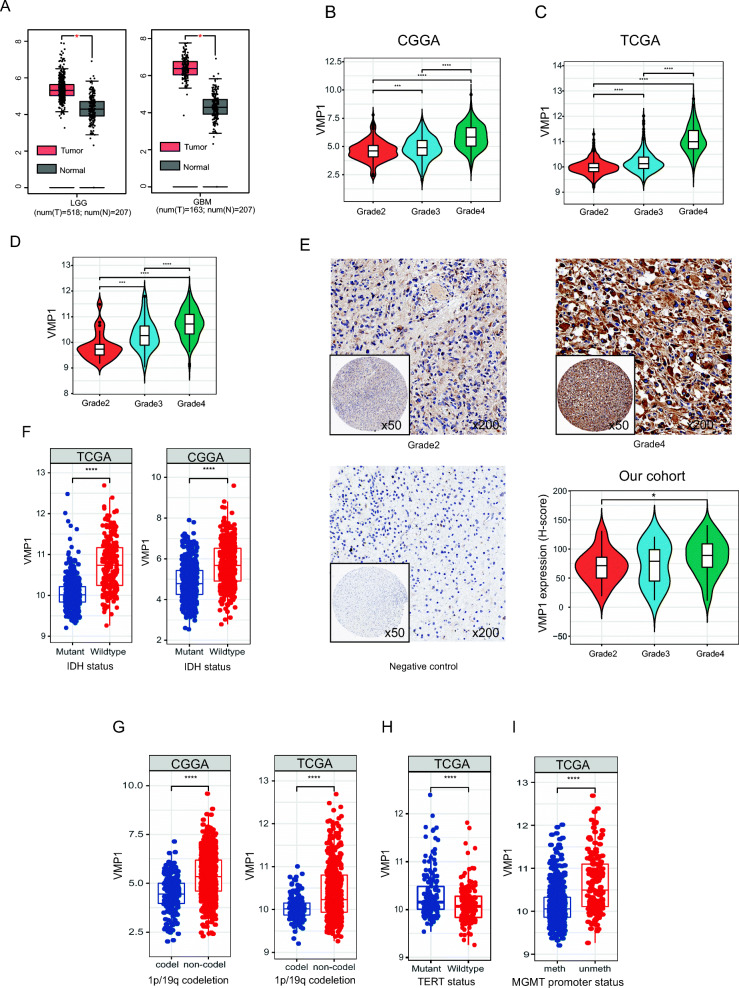
Associations between VMP1 expression and clinicopathological features. **A** Box diagram of *VMP1* gene expression in LGG, GBM, and normal tissues. Violin plot of *VMP1* gene expression for grades 2–4 in TCGA (**B**), CGGA (**C**), and GSE16011 (**D**) datasets. The black horizontal line represents the median gene expression level for each grade, and the upper and lower edges in the white box represent the upper and lower quartiles in the data set. The violin graph can also reflect the data density, where a more concentrated data set yields a broader graph. **E** Immunohistochemical analysis of VMP1 protein expression for different grades. Box diagram of *VMP1* gene expression with respect to different clinical pathological features, including the IDH status (**F**), 1p/19q codeletion (**G**), TERT status (**H**), and MGMT promoter status (**I**). P-values were determined using Wilcoxon tests (*P < 0.05; ***P < 0.001; ****P < 0.0001)

Data from TCGA and GTEx databases revealed that VMP1 was overexpressed in the lower grade glioma (LGG; *n* = 518) and GBM sample (*n* = 163) compared with the normal brain tissue (*n* = 207) (Fig. 1a). We next analyzed associations between VMP1 expression and clinical pathological parameters, including the tumor grade, isocitrate dehydrogenase (IDH) status, 1p/19q codeletion, TERT status, and O^6^-methylguanine DNA methyltransferase (MGMT) promoter status. As shown in Fig. 1b-d, *VMP1* mRNA levels showed a gradual increase from grade 2 to grade 4 using data from three databases (TCGA, CGGA, and GEO). Consistent with these results, an immunohistochemical analysis confirmed that high protein levels of VMP1 are markedly associated with a high grade (Fig. 1e). Additionally, high VMP1 expression was also associated with IDH wild-type (Fig. 1f), 1p/19q codeletion (Fig. 1g), TERT mutation (Fig. 1h), and MGMT promoter unmethylation (Fig. 1i).

Together, these findings indicate that *VMP1* may act as an oncogene that promotes glioma progression.

### VMP1 overexpression predicts a poor prognosis in glioma

**Fig. 2:**
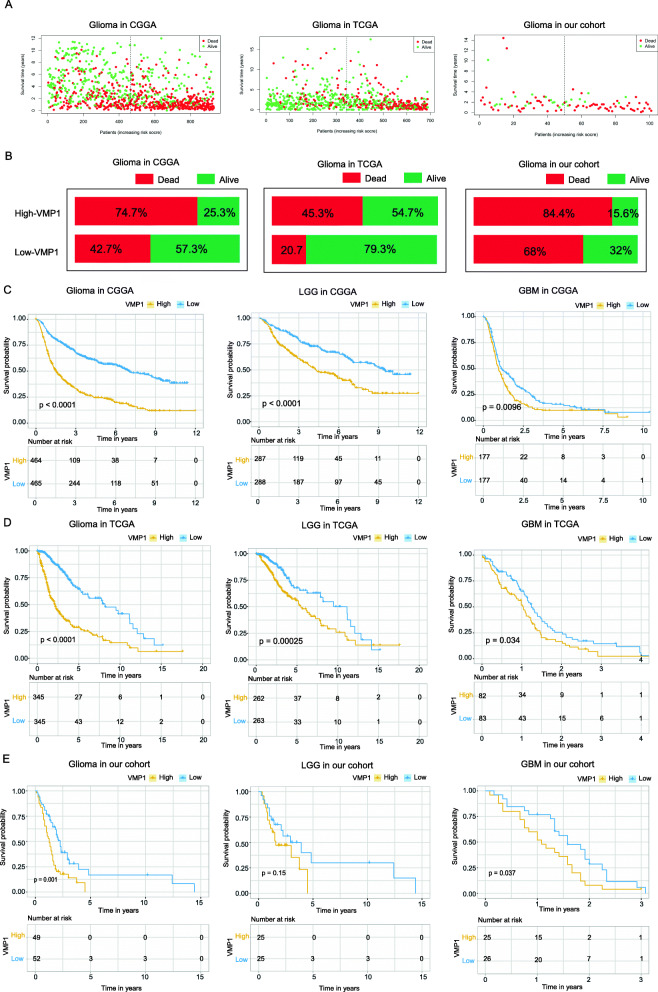
Prognostic value of VMP1 expression in glioma. **A** Patient status distributions in the high and low VMP1 expression groups. Each dot shows the patient status ranked by increasing expression. The *X*-axis shows the number of patients and the *Y*-axis shows the survival time. **B** Mortality rates in the high and low VMP1 expression groups. Kaplan–Meier overall survival curves for patients assigned to each VMP1 expression group in three datasets: CGGA (**C**), TCGA (**D**), and cohort (**E**)

VMP1 is associated with advanced glioma; therefore, we investigated the prognostic value of VMP1 in three independent cohorts. The mortality rate in the VMP1 high-expression group was significantly higher than that in the low-expression group (Fig. 2a, b). A Kaplan–Meier test showed that high VMP1 expression levels are markedly associated with a poorer OS in glioma. Next, a subgroup analysis of WHO grades revealed that patients with high VMP1 expression had a significantly shorter OS than patients with low VMP1 expression in LGG and GBM (Fig. 2c). These results were further confirmed in the CGGA dataset and our cohort (Fig. 2d, e).

**Fig. 3:**
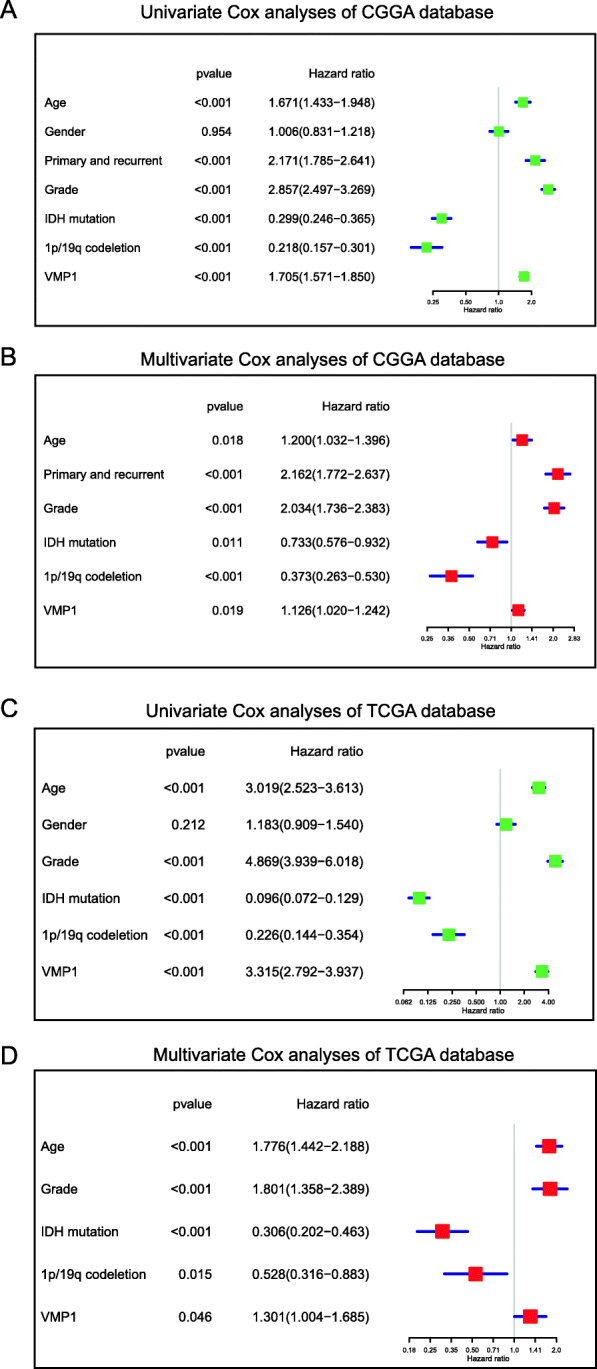
VMP1 expression serves as an independent prognostic factor. **A** Univariate Cox analyses revealed significant prognostic parameters. **B** Multivariate Cox analyses identified independent prognostic factors. **C**, **D** The results of univariate Cox analyses and multivariate Cox analyses were further validated in TCGA

Univariate and multivariate Cox analyses were performed to estimate the independent prognostic value of VMP1 in glioma. The univariate analysis revealed that high VMP1 expression was significantly associated with a poor OS (HR = 1.70, 95% CI 1.57–1.85, *P* < 0.01; Fig. 3a). Other variables associated with survival included age, WHO grade, primary and recurrent disease type, IDH status, and 1p/19q status. In multivariate analysis, high VMP1 expression was independently related with a poorer OS in glioma (HR = 1.12, 95% CI 1.02–1.24, *P* = 0.01; Fig. 3b). These results were validated using data obtained from TCGA (Fig. 3c, d).

Overall, these results demonstrate that VMP1 expression is significantly associated with prognosis in glioma and could be exploited as a biomarker for predicting survival.

### *VMP1* knockout (KO) by the CRISPR-Cas9 gene editing system significantly inhibits cell proliferation

**Fig. 4:**
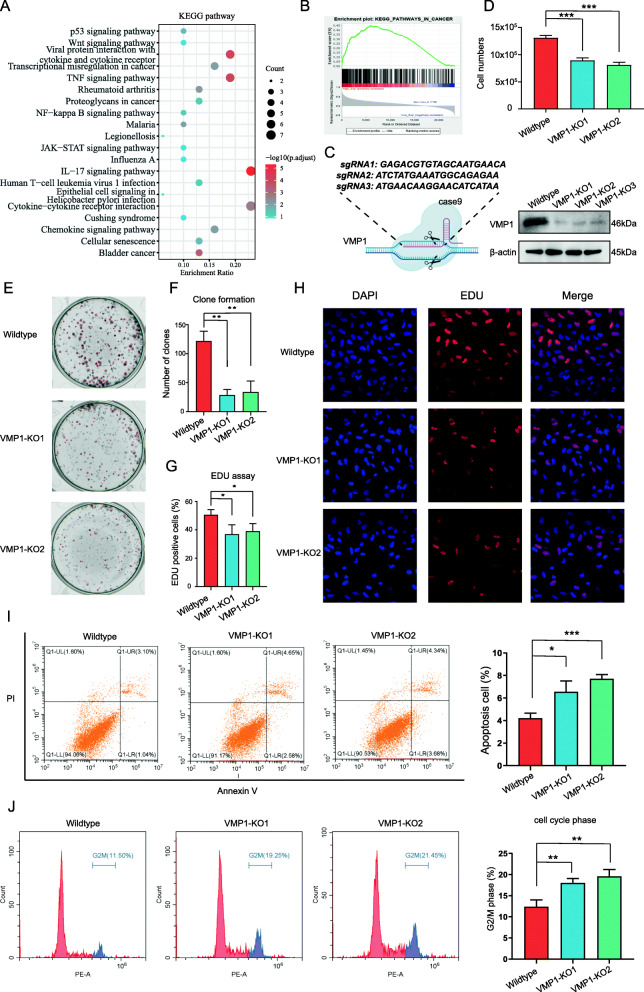
VMP1 knockout inhibits cell proliferation and induces cell death. **A** A KEGG signaling pathway enrichment analysis of differentially expressed genes (DEGs) between the VMP1 high and low groups. The intensity of the color represents the − log10 adjusted P-value, and dot sizes show the number of DEGs enriched in the corresponding pathway. **B** GSEA revealed that genes in the VMP1 high group were enriched in cancer hallmark pathways. Gene sets with normalized enrichment score (NES) > 1 and nominal *P*-value (NOM P-val) < 0.05 were considered significant. **C** Schematic diagram illustrating the CRISPR-Cas9-mediated VMP1 editing system and western blotting to confirm the decrease in VMP1 expression. **D** Cell growth in the wild-type group, VMP1 sgRNA1 group (VMP1 KO1), and VMP1 sgRNA2 group (VMP1 KO2). **E**, **F** Results of a clonogenic assay. Red circles represent the clones being counted. **G**, **H** Visualization of DNA replication by EdU incorporation. Cell nuclei stained in red represent DNA replication. **I** Annexin V-FITC and PI staining to determine the percentage of apoptotic cells. **J** Cell cycle analysis. P-values were determined via Student’s *t*-test (*P < 0.05; **P < 0.01; ***P < 0.001)

To elucidate the biological functions of VMP1 in tumorigenesis and progression and the signaling pathways underlying these functions, we conducted systematic Kyoto Encyclopedia of Genes and Genomes (KEGG) analyses and GSEA of DEGs between the VMP1 high-expression and low-expression groups. DEGs upregulated in the VMP1 high-expression group were enriched in processes related to tumor proliferation, such as the p53, NF-kappa B, and JAK−STAT signaling pathways (Fig. 4a). Consistent with the results of the KEGG pathway analysis, GSEA confirmed that cancer-related pathways were enriched in the VMP1 high-expression group, further supporting the potential tumorigenic effect of VMP1 (Fig. 4b).

To further validate the functions of VMP1, we used the CRISPR-Cas9 gene editing system for VMP1 depletion in the glioma cell line LN299. As shown in Fig. 4c, three sgRNAs were designed to knock down VMP1 expression. The protein level of VMP1 standardized to β-actin was apparently reduced by the transfection with each of the sgRNAs, as demonstrated by western blotting (Fig. 4c).

Cell proliferation was assessed via (i) cell counts, (ii) a clone formation assay, and (iii) an EdU incorporation assay. The cell counts were decreased in the two independent VMP1 KO groups after 48 h of incubation (Fig. 4d). Similarly, the clone formation and EdU incorporation assays revealed the attenuation of proliferation and DNA replication in the VMP1-KO groups (Fig. 4e-h). VMP1 KO significantly increased apoptosis and induced G2/M phase cell cycle arrest (Fig. 4i, j).

These results indicate that VMP1 is essential for tumorigenesis and the progression of glioma.

### *VMP1* depletion blocked the progression of autophagic flux

**Fig. 5:**
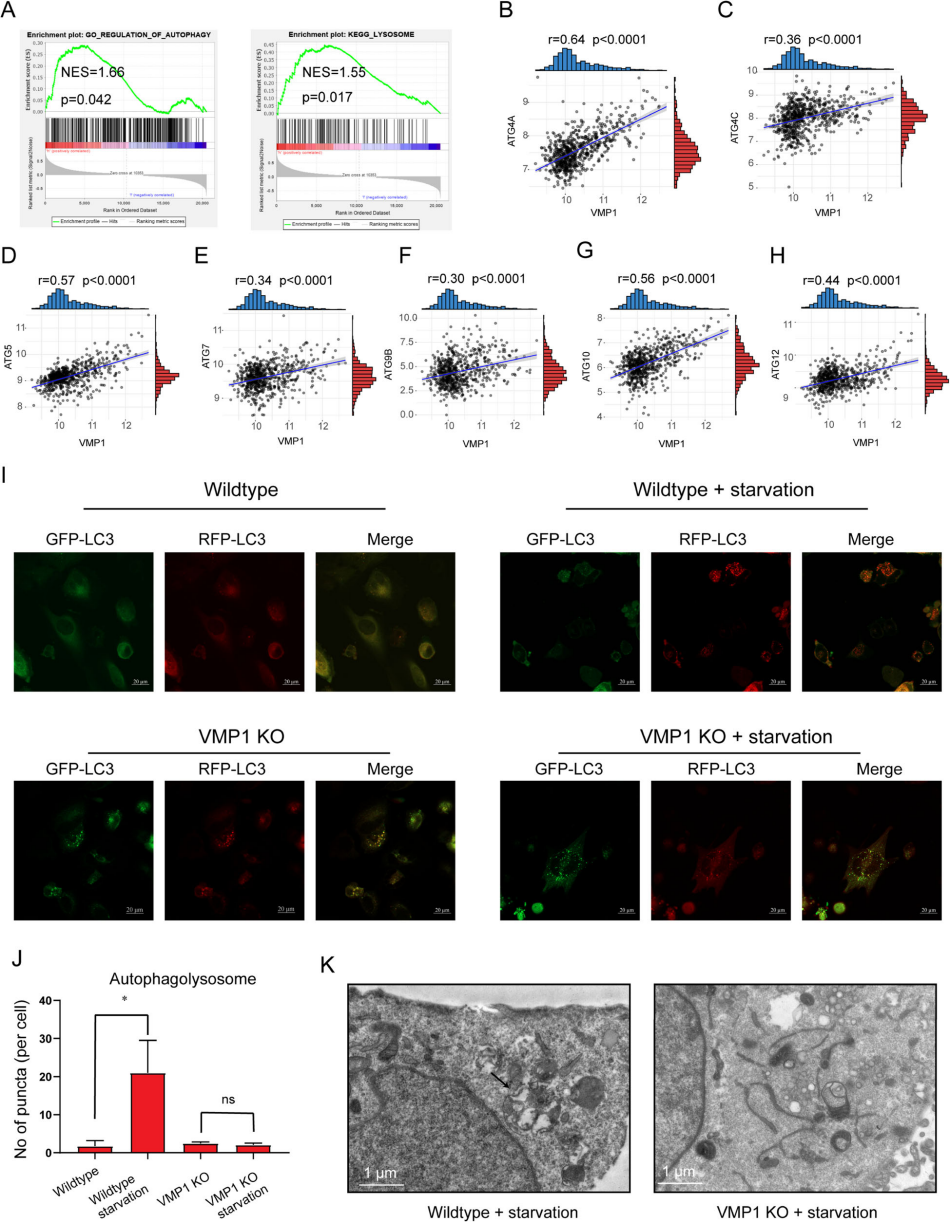
*VMP1* depletion inhibits the progression of autophagic flux. **A** GSEA showed that genes in the VMP1 high group were enriched in pathways related to the regulation of autophagy and lysosomes. Correlation between VMP1 expression and autophagy-related genes, including *ATG4A* (**B**), *ATG4C* (**C**), *ATG5* (**D**), *ATG7* (**E**), *ATG9B* (**F**), *ATG10* (**G**), and *ATG12* (**H**). **I**, **J** RFP-GFP-LC3B dual-reporter assay showing the accumulation of autolysosomes. RFP-positive and GFP-negative dots indicate autolysosomes. **K** TEM microphotographs showing the ultrastructure of cells. Red arrows indicate endogenous autolysosomes

Autophagy is associated with the maintenance of cell homeostasis and has therefore received tremendous attention in cancer research. Previous studies have demonstrated that VMP1 plays a critical role in maintaining autophagic flux. We hypothesized that VMP1 contributes to glioma progression by modulating autophagy. Indeed, GSEA revealed enrichment for the regulation of autophagy and lysosomes in TCGA (Fig. 5a). VMP1 expression was positively correlated with levels of autophagy-related genes, including *ATG4A*, *ATG4C*, *ATG5*, *ATG7*, *ATG9B*, *ATG10*, and *ATG12* (Fig. 5b-h).

The fusion of autophagosome-lysosome and autolysosome acidification comprises late steps in the autophagic process, which is the key to maintain functional autophagic flux and cellular homeostasis. We assessed autophagic flux via a dual red fluorescent protein (RFP)-green fluorescent protein (GFP)-LC3B puncta assay and TEM. In the RFP-GFP-LC3 dual-reporter system, LC3B was tandemly tagged with acid-sensitive GFP and acid-resistant monomeric RFP to assess the formation of autophagosomes (RFP+ GFP+ signal) and autolysosomes (RFP+ GFP− signal). As shown in Fig. 5i and j, autolysosomes (RFP+ GFP− signal) were significantly elevated under starvation. In contrast, in VMP1-KO cells, autolysosomes (RFP+ GFP− signal) were not reduced under starvation, suggesting that autophagic flux was almost completely blocked. TEM indicated the noticeable accumulation of double-membrane electron-dense autolysosomes under starvation in the wild-type group but not in the VMP1-KO group (Fig. 5k). These findings demonstrated that autophagic flux is blocked in VMP1-KO cells.

### *VMP1* depletion sensitizes glioma to radiotherapy and chemotherapy

**Fig. 6:**
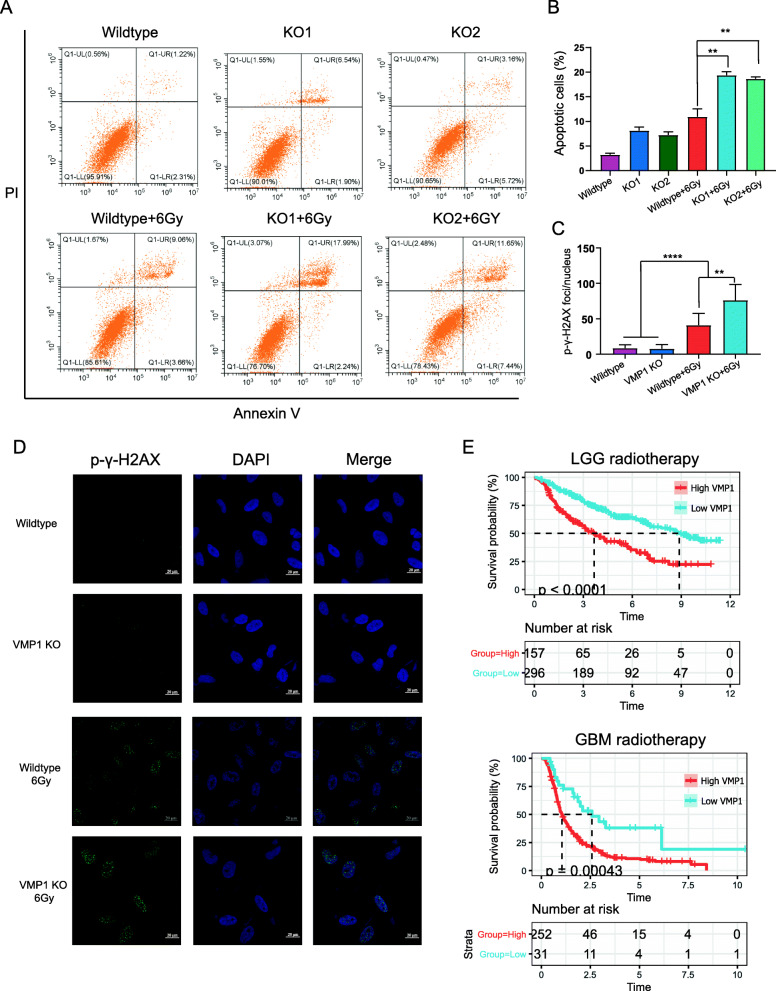
*VMP1* depletion sensitizes glioma to radiotherapy. **A**, **B** Annexin V-FITC and PI staining using flow cytometry. **C**, **D** Quantification of the formation of phosphorylated (S139) γ-H2AX foci, as determined by immunofluorescence. **E** Kaplan–Meier overall survival curves for patients in the VMP1 high and low groups who received radiotherapy. P-values were determined by Student’s *t*-tests (*P < 0.05; **P < 0.01; ***P < 0.001; ****P < 0.0001)

Autophagy is commonly defined as a cell survival mechanism and is associated with the resistance to radiotherapy and chemotherapy. Under different stimuli, including radiotherapy and chemotherapy, moderate autophagy is a normal metabolic process involved in the removal of damaged proteins and organelles and provides additional energy during stress. Due to the protective function of autophagy, inhibitors of this process and the silencing of autophagy-related genes have the potential to increase tumor cell radiosensitivity and chemosensitivity. We demonstrated that VMP1 KO could promote radiation-induced apoptosis under 6 Gy of radiation in the LN299 cell line (Fig. 6a, b). Additionally, p-γ-H2AX, a marker of DNA double-strand breaks (DSBs), was analyzed to further assess the effect of VMP1 KO and radiation on DNA damage. As evidenced by the formation of p-γ-H2AX foci, VMP1 KO significantly increased radiation-induced DSBs (Fig. 6c, d). In addition, in the CGGA database, among patients receiving radiotherapy (radiotherapy alone and combined treatment), survival was longer in the VMP1 low-expression group than in the VMP1 high-expression group (Fig. 6e).

**Fig. 7:**
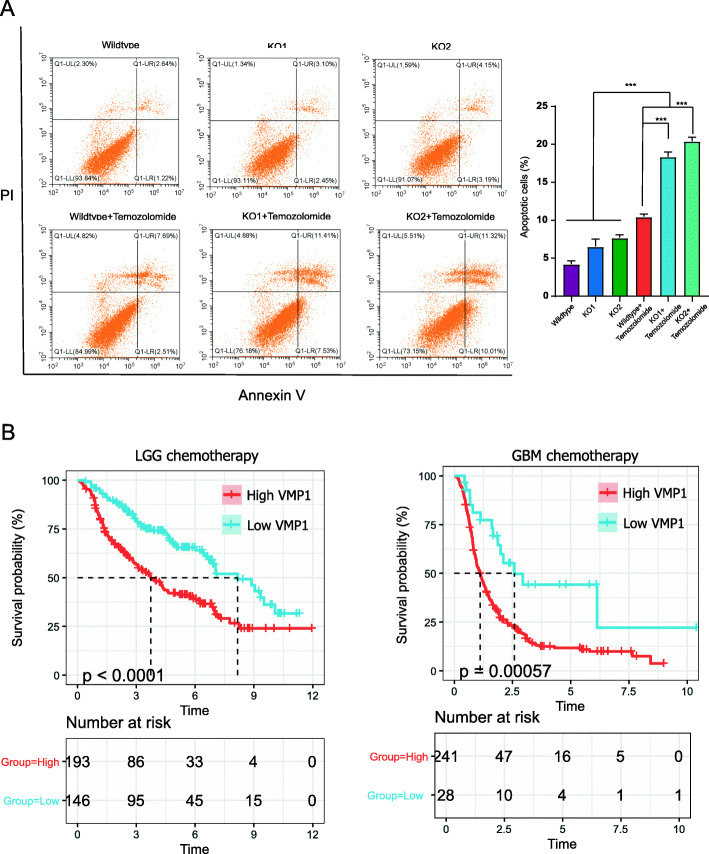
VMP1 depletion sensitizes glioma to chemotherapy. **A** Annexin V-FITC and PI staining using flow cytometry. **B** Kaplan–Meier overall survival curves for patients in the VMP1 high and low groups who received chemotherapy. P-values were determined by Student’s *t*-tests (*P < 0.05; **P < 0.01; ***P < 0.001)

Similarly, VMP1 KO could significantly increase temozolomide-induced apoptosis under 200-μM concentration (Fig. 7a). Moreover, among patients receiving chemotherapy (temozolomide alone and combined treatment), survival was longer in the VMP1 low-expression group than in the high-expression group (Fig. 7b). The clinical characteristic of patients from CGGA cohort has uploaded as supplementary materials (see Additional file 1).

### Nomogram model based on VMP1 expression shows high prognostic value in glioma

**Fig. 8:**
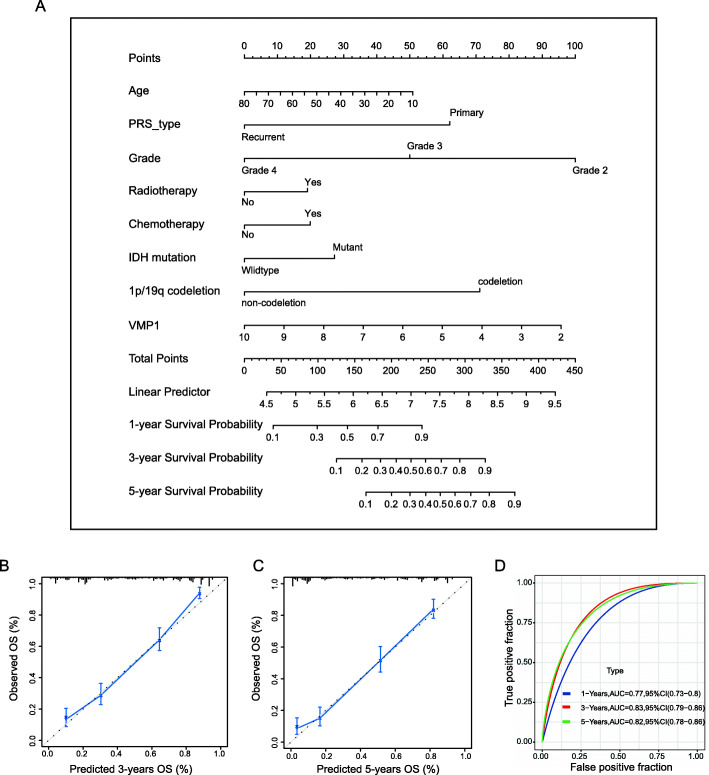
Nomogram development and performance. **A** Construction of a nomogram model to predict 1-, 3-, and 5-year OS based on VMP1 expression and clinicopathological parameters. Calibration plots of the nomogram for 3-year (**B**) and 5-year (**C**) survival. The *x*-axis shows the nomogram-predicted probability and the *y*-axis represents the observed probability. **D** ROC curve showing the predictive efficiency of the nomogram for 1-, 3-, and 5-year OS

Given the important biological functions of VMP1 in tumorigenesis and its association with clinicopathologic features, we further constructed a nomogram model in which VMP1 expression was integrated with seven clinical characteristics (age, primary recurrence type, grade, radiotherapy status, chemotherapy status, IDH mutation status, and 1p/19q codeletion status; Fig. 8a). To assess the predictive efficiency of the nomogram for the 3- and 5-year survival rates, we generated calibration plots and receiver operating characteristic (ROC) curves. Calibration plots for observed vs. predicted probabilities of 3- and 5-year OS showed excellent concordance (Fig. 8b), and the areas under the ROC curve were 0.77 at 1 year, 0.83 at 3 years, and 0.82 at 5 years, indicating a high predictive value (Fig. 8c).

## Discussion

We identified VMP1 as a novel oncogene in glioma. VMP1 was overexpressed in glioma and was associated with disease progression. Clinically, high VMP1 expression was independently associated with a dismal prognosis in glioma and could be exploited as a biomarker for predicting survival. VMP1 KO significantly inhibited glioma cell proliferation, induced apoptosis and cell cycle arrest, and sensitized glioma to radiotherapy and chemotherapy by disrupting autophagic flux.

VMP1 was originally characterized as a pancreatitis-associated protein [19]; it is a multi-spanning membrane protein in the ER and is critical for autophagosome formation in mammals [20]. When VMP1 is depleted, LC3 puncta accumulated and failed to separate from the ER membrane under both nutrient repletion and deprivation conditions, causing a dysfunctional autophagic flux [11]. Consistent with this, we observed the accumulation of LC3 puncta under both nutrient repletion and deprivation conditions, whereas autophagosome-lysosome formation was impaired in VMP1 KO glioma cells, resulting in incomplete autophagy.

Autophagy mediates resistance to radiotherapy or chemotherapy [21, 22]. Mechanistically, the autophagic processes enable cells to degrade misfolded/unfolded proteins and cytoplasmic organelles, such as damaged ribosomes and mitochondria, the products of which are recycled to generate macromolecules and ATP to maintain cellular homeostasis [23–26]. As a part of this process, intracellular vesicles termed autophagosomes envelope intracellular organelles and then fuse with lysosomes, wherein degradation occurs. In our research, VMP1 KO disrupted autophagosome formation and fusion with lysosomes, which reduced catabolites for recycling and inhibited energy metabolism, thus sensitizing glioma cells to radiotherapy and chemotherapy.

Recently, studies on the effect of VMP1 on autophagy have increased nearly exponentially; however, little is known regarding its diagnostic and prognostic value. In hepatocellular carcinoma and colorectal cancer, low VMP1 expression is associated with advanced cancer stage and short survival, and VMP1 downregulation confers an aggressive phenotype [27, 28]. However, the opposite results have been observed in ovarian cancer and acute myeloid leukemia, in which elevated VMP1 expression contributes to cancer progression and is associated with a poor prognosis [29, 30]. Consistent with results obtained for ovarian cancer and acute myeloid leukemia, our study of glioma revealed that VMP1 overexpression is related to disease progression and a poor prognosis, and VMP1 KO inhibits cell proliferation and induces cell death. These findings indicate that VMP1 may serve as a cancer-promoting factor in glioma.

GBM is categorized as the most malignant intracranial tumor (grade IV) that is highly aggressive and heterogeneous. Thus, the identification of accurate biomarkers and potential therapeutic targets for GBM is essential to improve disease outcome. In our study, VMP1 is identified as a novel oncogene and biomarker with a characterized cancer-promoting role in GBM development. It has been reported that VMP1 expression is enhanced in several cancers. Herein, we systematically evaluated VMP1 expression in different grade of glioma. Our results revealed that the expression of VMP1 presented the highest level in GBM, and shared a gradual decrease from the WHO grade III to WHO grade II, indicating that VMP1 might facilitate tumor progression. Indeed, in vitro experiments verified VMP1 depletion by the CRISPR-Cas9 gene editing system significantly inhibited GBM cell proliferation, induced cell cycle arrest, and promoted cell apoptosis, which further validated the oncogenic function of VMP1 in GBM. These results suggest that VMP1 may serve as a potential therapeutic target. Besides, survival analysis shows that patient with low VMP1 expression presented a better prognosis and a sensitivity to radiotherapy and chemotherapy, which provided potential hints for therapeutic options. Moreover, mechanistically, VMP1 is the critical upstream regulator that switches the autophagic flux and thus maintains cellular homeostasis. Although small molecule inhibitors targeting VMP1 have not been developed, autophagy inhibitors, such as chloroquine and hydroxychloroquine, have been clinically approved, promoting combination treatment for patients with high VMP1 expression. Nevertheless, additional studies on VMP1 inhibitor investigation are needed.

The autophagy-related protein VMP1 has been linked to the development and progression of cancer. In this study, we clearly establish the prognostic value of VMP1 in glioma based on bioinformatics and cell-based analyses. Furthermore, we show that VMP1 contributes to cancer development and progression via the regulation of autophagy. These results improve our understanding of the roles of VMP1 in tumor-related processes and suggest that it is a prognostic biomarker for glioma, a highly aggressive tumor type. However, it should be noted that the findings of this study have to be seen in light of some limitations. Although biological functions of VMP1 were proved by in vitro experiments, in vivo studies, and reverse experiment are missing. Further validation on reverse experiment and in vivo studies could contribute to confirm the relevance of VMP1 in glioma growth. Our findings should be interpreted with this limitation in mind.

## Conclusions

Our results provide evidence for the prognostic value of VMP1 in glioma. We found that VMP1 promotes tumor growth and progression and mediates resistance to chemotherapy and radiotherapy by manipulating autophagy.

## Data Availability

The datasets generated and/or analyzed during the current study are available in the TCGA and GEO repository (https://www.cancer.gov/tcga; https://www.ncbi.nlm.nih.gov/geo/).

## Abbreviations

ER: Endoplasmic reticulum
CGGA: Chinese Glioma Genome Atlas
DEGs: Differentially expressed genes
DSB: Double-strand break
GBM: Glioblastoma
GEO: Gene Expression Omnibus
GFP: Green fluorescent protein
GSEA: Gene set enrichment analysis
HRP: Horseradish peroxidase
IDH: Isocitrate dehydrogenase
KEGG: Kyoto Encyclopedia of Genes and Genomes
LC3: Light chain 3
LGG: Low-grade glioma
MGMT: O^6^-methylguanine DNA methyltransferase
OS: Overall survival
PBS: Phosphate-buffered saline
PI: Propidium iodide
RFP: Red fluorescent protein
ROC: Receiver operating characteristic
sgRNA: Small guide RNA
TCGA: The Cancer Genome Atlas
TEM: Transmission electron microscopy
VMP1: Vacuole membrane protein 1
